# Supplementary material for: Isolation of Novel Xanthomonas Phages Infecting the Plant Pathogens X. translucens and X. campestris
Source: Viruses. 2022 Jun 30;14(7):1449. doi: 10.3390/v14071449 (PMC9316219; doi:10.3390/v14071449)
Supplement: Supplementary file 1 [file viruses-14-01449-s001.zip › viruses-1754070-supplementary.pdf]

Supplementary Information to

Isolation of novel *Xanthomonas* phages for the plant pathogens *X. translucens* and *X. campestris*

Sebastian H. Erdrich [1], Vikas Sharma [2], Ulrich Schurr [1], Borjana Arsova [1] and Julia Frunzke [2]*

[1] Institute of Bio- and Geosciences, Department for Plant Sciences (IBG-2), Forschungszentrum Jülich, 52425 Jülich, Germany
[2] Institute of Bio- and Geosciences, Department for Biotechnology (IBG-1), Forschungszentrum Jülich, 52425 Jülich, Germany; s.erdrich@fz-juelich.de (S.E.); v.sharma@fz-juelich.de (V.S.); b.arsova@fz-juelich.de (B.A); u.schurr@fz-juelich.de (U.S)
* Correspondence: j.frunzke@fz-juelich.de; Tel.: +49-2461-615430

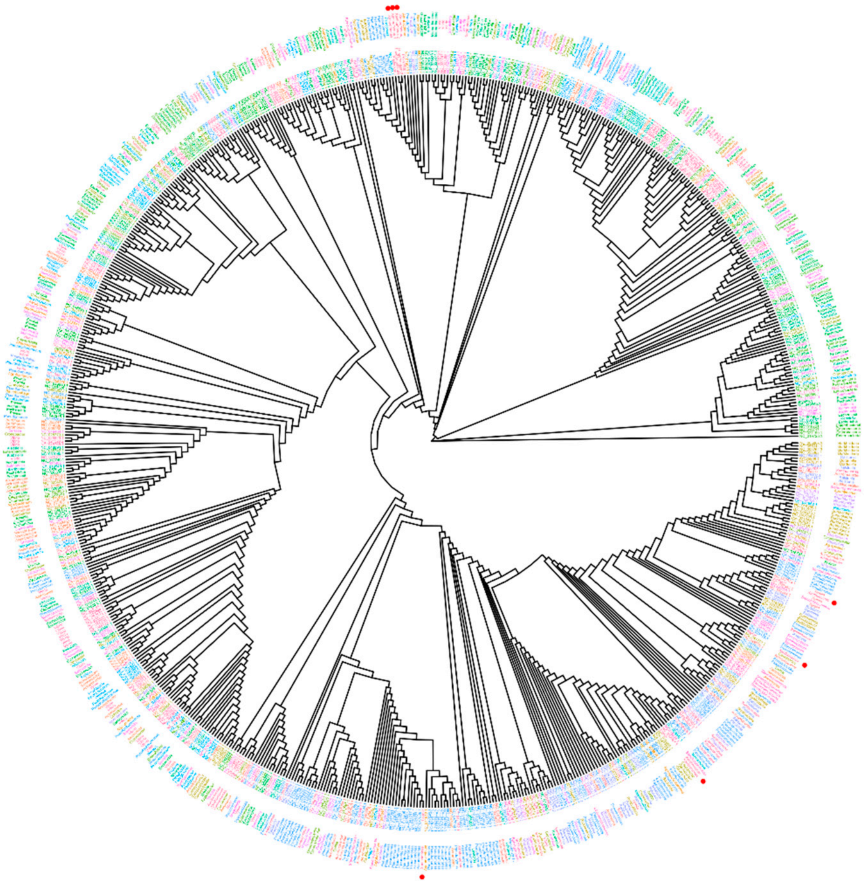

Figure S1. K-mer clustering dendrogram of Proteobacteria phages. More than 2000 Phage genomes were retrieved from VirusHost DB [29] filtered by host genus. Afterwards a clustering with 90% identity level was performed, the remaining 725 phages, as well as the closest relatives to our phages (red dots) according to NCBI Blast were used to build the dendrogram.

	Langgrundblatt1	Langgrundblatt2	Pfeifenkraut	Elanor	Laurilin	Mallos	Seregon
Xanthomonas translucens	1	1	1	1	1	1	0.000004
Xanthomonas campestris	0.00002	0.0003	0.00007			0.00002	1
Sinorhizobium meliloti							
Bacillus subtills							
Herbaspirillum seropedicae							
Azospirillum brasilense sp245							
Pseudomonas flourecence						0.00004	
Pseudomonas koreensis							
Pseudomoas syringae pv lapsa							
Pseudomoas syringae pv tomato						0.00002	
Agrobacterum tumefaciens							

Figure S2. Host range assay. The host range of the seven phages was determined by spotting serial dilutions of the phages on lawns of different Xanthomonads, plant pathogenic bacteria and plant growth promoting bacteria (PGPB) propagated on the respective medium. Lysis is indicated as follows; plaque formation on isolation host (dark green), plaque formation on other bacteria or clearance of the lawn (light green), no plaques or lysis visible (no color). Numbers indicate the efficiency of plating (EOP).

Table S1. Phage particle size. Measurements of virion particles analyzed by Transmission electron microscopy (TEM).

Phage Name.	Capsid diamaeter	Tail length	Phage size
Langgrundblatt 1	56 nm ± 3	170 nm ± 8	226 nm
Langgrundblatt 2	57 nm ± 3	160 nm ± 18	217 nm
Pfeifenkraut	55 nm ± 3	153 nm ± 10	208 nm
Elanor	77 nm ± 5	134 nm ± 12	211 nm
Laurelin	77 nm ± 2	143 nm ± 7	220 nm
Mallos	82 nm ± 2	141 nm ± 7	223 nm
Seregon	66 nm ± 3	225 nm ± 7	291 nm

Table S2. Strains /Phages used in this study.

Organism	Source	Reference
Xanthomonas translucens (DSM 18974)	DSMZ	https://www.dsmz.de/collection/catalogue/details/culture/DSM-18974
Xanthomonas campestris pv. *campestris*	AG Narberhaus (RUB, Bochum)	[14]
Sinorhizobium meliloti 1021 (DSM 30135)	DSMZ	https://www.dsmz.de/collection/catalogue/details/culture/DSM-30135
Bacillus subtills (DSM 10)	DSMZ	https://www.dsmz.de/collection/catalogue/details/culture/DSM-10
Herbaspirillum seropedicae (DSM 6445)	DSMZ	https://www.dsmz.de/collection/catalogue/details/culture/DSM-6445
Azospirillum brasilense sp245	AG Arsova	[51]
Pseudomonas fluorescence (DSM 50090)	DSMZ	https://www.dsmz.de/collection/catalogue/details/culture/dsm-50090
Pseudomonas koreensis (DSM 16610)	DSMZ	https://www.dsmz.de/collection/catalogue/details/culture/DSM-16610
Pseudomoas syringae pv *lapsa* (DSM 50274)	DSMZ	https://www.dsmz.de/collection/catalogue/details/culture/DSM-50274
Pseudomoas syringae pv *tomato* DC3000	AG Ott (University Freiburg)	[52]
Agrobacterum tumefaciens C58	AG Narberhaus (RUB, Bochum)	[53]
